# Selective Multiphase‐Assisted Oxidation of Bio‐Sourced Primary Alcohols over Ru‐ and Mo‐ Carbon Supported Catalysts

**DOI:** 10.1002/cssc.202400888

**Published:** 2024-11-08

**Authors:** Chiara Bersani, Daily Rodríguez‐Padrón, Daniel Ballesteros, Enrique Rodríguez‐Castellón, Alvise Perosa, Maurizio Selva

**Affiliations:** ^1^ Dipartimento di Scienze Molecolari e Nanosistemi Università Ca' Foscari Venezia Via Torino 155 30175 Venezia Mestre Venice Italy; ^2^ Department of Inorganic Chemistry Facultad de Ciencias Universidad de Málaga Campus de Teatinos s/n 29071 Málaga Spain

**Keywords:** Multiphase systems, heterogeneous catalysis, alcohols oxidation, Ionic liquids, Catalyst recyclability

## Abstract

The oxidation of representative bio‐based benzyl‐type alcohols has been successfully carried out in a multiphase (MP) system comprised of three mutually immiscible liquid components as water, isooctane, and a hydrophobic ionic liquid as methyltrioctylammonium chloride ([CH_3_(CH_2_)_6_CH_2_]_3_N(Cl)CH_3_), a heterogeneous catalyst (either *ad‐hoc* synthesized carbon‐supported Mo or a commercial 5 % Ru/C), and air as an oxidant. The MP‐reaction proceeded as an interfacial process with Mo/C or Ru/C perfectly segregated in the ionic liquid phase and the reactant(s)/products(s) dissolved in the aqueous solution. This environment proved excellent to convert quantitatively benzyl alcohols into the corresponding aldehydes with a selectivity up to 99 %, without overoxidation to carboxylic acids. The nature of the catalyst, however, affected the operating conditions with Ru/C active at a lower T and t (130 °C, 4–6 h) compared to Mo/C (150 °C, 24 h). The phase confinement was advantageous also to facilitate the products isolation and the recycle of the catalyst. Notably, in the Mo/C‐catalyzed oxidation of benzyl alcohol, benzaldehyde was achieved with unaltered selectivity (>99 %) at complete conversion, for five subsequent reactions through a semicontinuous procedure in which the catalyst was reused *in‐situ*, without ever removing it from the reactor or treating it in any way.

## Graphical abstract

Multiphase‐assisted strategy for fine‐tuning the selectivity of biomass‐derived primary alcohol oxidation, while ensuring catalyst recyclability and reusability.

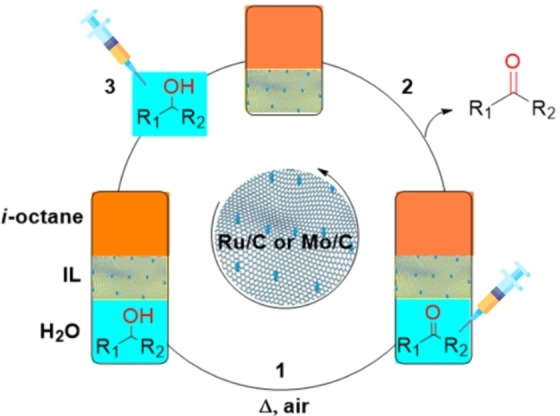

## Introduction

**Figure 1:**
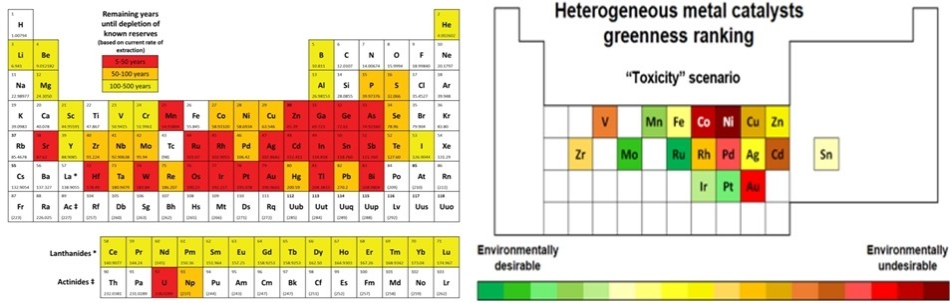
Left: remaining years until depletion of known reserves of elements (reproduced from ref. [7]); right: ranking of heterogeneous metal‐based catalysts by their greenness (reproduced from ref. [8]).

Heterogenous catalysis is fundamental to most diverse society needs, from the chemicals and polymers synthesis to the energy and waste management, e‐chemistry (fossil fuel‐free) transition, and more.[^1^, ^2^] In response to this global demand, the design of catalysts has become so sophisticated that the properties of such systems, including stability and reuse over time, are currently highly reliable and reproducible.^[3]^ Just think for example, to heterogeneous single‐cluster catalysts (SCCs) that promise to disclose a new frontier for complex and technically relevant reactions.^[4]^ The selection of a catalyst for a given process, however, is neither obvious nor straightforward. Issues and criticalities concern the use of endangered metals, particularly Pt, Pd, Ir, Os, Rh and Ru which are consumed by more than 90 % in the electronics and catalyst manufacturing,^[5]^ the geopolitical instability of areas that provide such elements and the volatility of the corresponding markets,^[6]^ and their release in the planet ecosystems. Interesting studies have been proposed to classify metals by both their risk of depletion and ecofriendly character (Figure 1).[^7^, ^8^]

Albeit at the date of the study (2015), the depletion rate could not foresee the future development of recycling technologies nor the discovery of new reserves, certain non‐noble metals resulted far more sustainable over precious ones (Figure 1, left: compare yellow, ochre, and red boxes). On the other hand, a multicriteria evaluation with the TOPSIS methodology, demonstrated that ruthenium, iron, and molybdenum systems were the most favourable alternatives from the standpoint of toxicity, release, and life cycle assessment (LCA)‐related parameters (Figure 1, right).

These concepts have been largely transposed by the recent literature on heterogeneous catalysis, where hundreds of studies have been aimed to compare the performance and the environmental impact of non‐noble metal supported systems with respect to their noble counterparts.[^9^, ^10^, ^11^, ^12^, ^13^] An attractive field in this vast scenario deals with catalytic oxidations. We recently contributed this subject by designing a library of catalysts comprised of core‐shell nanoparticles of metal/metal oxides based on nickel, iron, cobalt and molybdenum supported on *N*‐doped carbons, that proved effective for the oxidation of different primary alcohols.^[14]^ In the presence of air as an oxidant, supported Mo was the most promising system to control the products distribution and achieve conversion and selectivity both above 90 % towards the corresponding aldehydes as products of partial oxidation. Results were satisfactory, yet the procedure required both a harmful solvent (acetonitrile) to ensure oxygen solubility and a costly/time‐consuming catalyst/product separation and catalyst recycle. These aspects prompted us to reconsider the oxidation processes by designing different reaction conditions, in a multiphase (MP) system. MP‐assisted protocols for catalytic organic synthesis have been widely investigated by our group, especially procedures based on combinations of two or more immiscible liquids such as water, hydrophobic ionic liquids (ILs), and organic solvents, have been explored.[^15^, ^16^, ^17^, ^18^] The multiple advantages of the MP‐methods include: i) the built‐in separation of reagents/products/catalyst; ii) the tuning of the reaction kinetics and the selectivity thanks to the changes in solvation and adsorption occurring at the liquid‐liquid interphases; iii) the segregation of the catalyst in a phase different from that where the reaction takes place; iv) the catalyst reuse which improves sustainability through process intensification and steps economy, and minimizes waste and energy/mass consumption.

In the current work, most, if not all, the i)‐iv) characteristics have been exploited to develop an innovative methodology for the oxidation of primary benzyl‐type alcohols under batch conditions. A MP‐environment, never previously explored for such reaction, was designed using two immiscible liquids as water and isooctane, with and without a hydrophobic ionic liquid as methyltrioctylammonium chloride ([CH_3_(CH_2_)_6_CH_2_]_3_N(Cl)CH_3_) as a third liquid component, was designed. The reaction occurred in the aqueous solution in the presence of air as the cheapest available oxidant, while a heterogeneous catalyst was segregated either in the isooctane or in the ionic liquid phase. An *ad‐hoc* synthesized carbon‐supported Mo (6.1 wt %) and a commercial 5 wt % Ru/C were the catalysts. The latter were chosen with the aim to include environmentally desirable metals, active for oxidation reactions, and in the case of Mo, a non‐noble element not in danger of extinction.

**Scheme 1:**
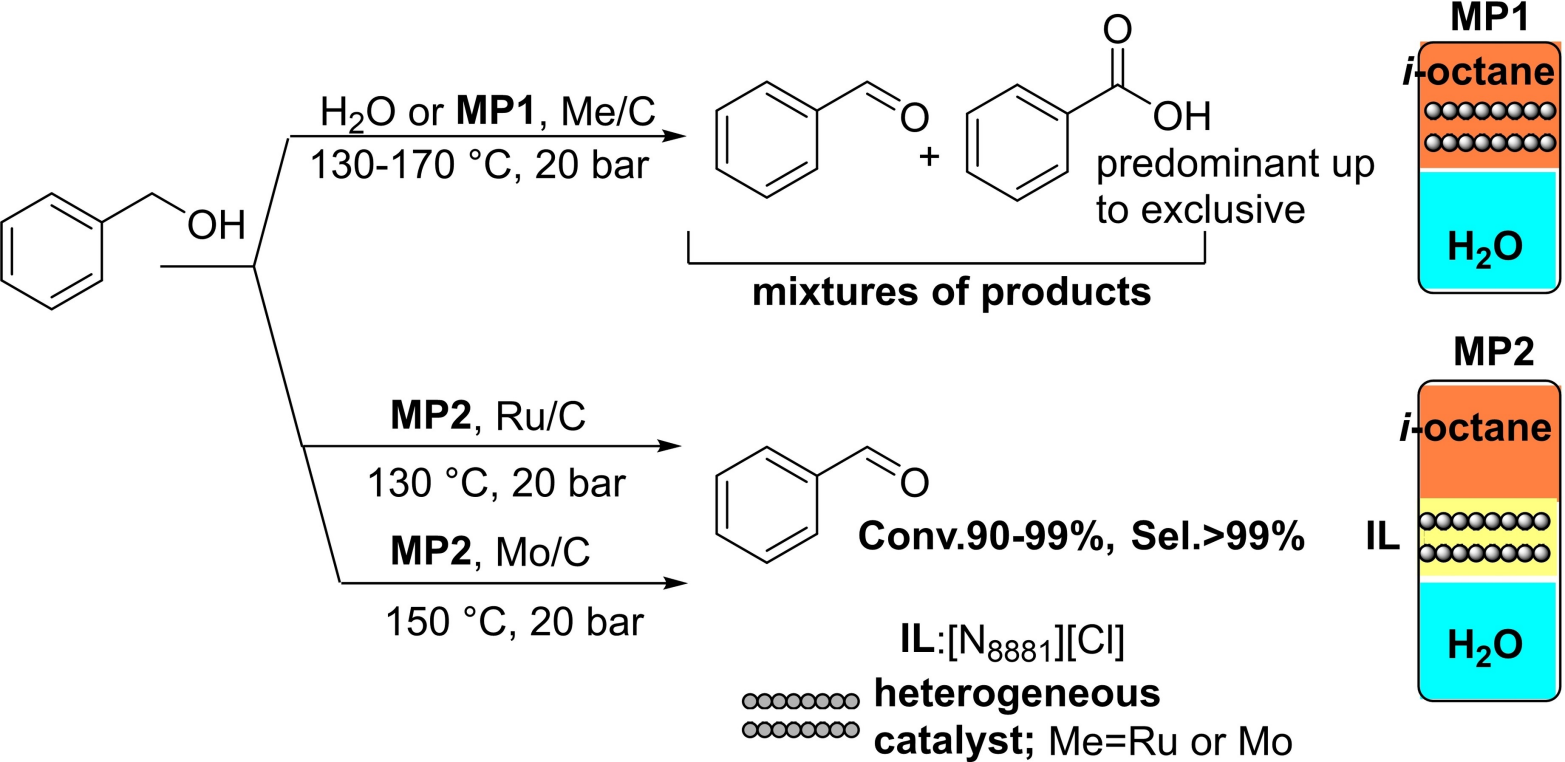
The oxidation of benzyl alcohol under multiphase conditions (MP1 and MP2) and in water, in the presence of Mo/C or Ru/C catalysts.

MP‐conditions offered an original unprecedented arrangement to control the reaction selectivity; particularly, the IL‐assisted process allowed to achieve only partially oxidized derivatives as aldehydes, notwithstanding the marked propensity of such products towards overoxidation to carboxylic acids. Scheme 1 showcases the reaction of benzyl alcohol as a model oxidation process.

In water or in the water/isooctane biphase (**MP1**), mixtures of products were generally achieved, and if experiments were prolonged up to 12–24 h, benzoic acid was obtained as an exclusive derivative (>99 %; top). By contrast, the ionic liquid assisted multiphase system (**MP2**) proved excellent to isolate benzaldehyde in a >99 % selectivity, at conversion above 90 % (bottom). The products distribution was mostly determined by the choice of the reaction environment (H_2_O, **MP1** or **MP2**), but the nature of catalyst affected the operating conditions with Ru/C active at a lower T and t (130 °C, 4 h) compared to Mo/C (150 °C, 24 h). Furthermore, a perfect products/catalyst separation was achieved using both **MP1** and **MP2**: products were confined in the polar media (water) and the catalyst was segregated in the hydrocarbon (isooctane) or the ionic liquid phase where it was recycled (coloured boxes on right). Changes in the properties of Mo/C after its use were thoroughly documented across a variety of characterization analyses. However, despite these transformations, the activity and selectivity of the catalyst were preserved over five subsequent reuses. This remarkable resilience due to the catalyst′s confinement not only ensured practical reusability but prevented any metal contamination of the product or loss of metal into the water phase.

Similar results were obtained also with other benzyl‐type alcohols bearing *p*‐Br, *p*‐OCH_3_, *p*‐OH, *o*‐OH or *m*‐OCH_3_‐*p*‐OH as aryl substituents. Regardless of conditions and catalysts, the oxidation of aliphatic alcohols was far less selective. These substrates were not only overoxidized to the corresponding carboxylic acids, but they underwent a competitive C−C bond cleavage process, yielding shorter chain derivatives. *n*‐Butyl alcohol, for example, was quantitatively converted to a mixture of butanoic, propanoic and acetic acids in a 70–75 %, 20–23 % and 3–6 %, respectively.

## Results and Discussion

### Catalytic Materials

#### Ru/C

C‐supported Ru is one the best options among heterogeneous catalysts for processing water‐soluble organic reactants, not only for its activity but also for its moderate cost which is about 4 % compared to that of other precious metals such as Au and Pt.[^19^, ^20^, ^21^, ^22^] Previous papers of our group proved that Ru/C‐based systems were excellent for a variety of multiphase processes (e. g., hydrogenation and reductive aminations of levulinic acid,^[17]^ hydrogenation of sugars,^[23]^ oxidation of HMF^[16]^) since they were effective catalysts and at the same time, they were hydrophobic enough to act in a phase, usually a hydrocarbon or an ionic liquid one, separated from that where reaction occurred, typically an aqueous solution (see Scheme 1). These reasons along with the easy commercial availability prompted us to choose a 5 % Ru/C sample sourced by Aldrich, as a benchmark catalyst for this study. The characterization of this system (lot# MKBW5890 V) for its structural, morphological, and acid properties was reported elsewhere.[^16^, ^17^]

#### Mo/C

Mo‐based materials have been largely explored in the literature as non‐noble metal alternatives for catalytic oxidations.[^24^, ^25^, ^26^] One of the most famous examples is the formox process for the conversion of methanol into formaldehyde where either the original catalyst (iron molybdate on excess molybdenum oxide) or innovative systems as supported molybdenum oxide on hydroxyapatites offer excellent conversions and selectivities, both above 90 %.[^27^, ^28^] This result and more generally, the catalytic behaviour of Mo in oxidation processes were explained also by theoretical studies that highlighted the role of the partially filled metal d‐band in enhancing the metal affinity for reactants or adsorbates.^[29]^ In this paper, a carbon‐supported Mo‐based material was designed to achieve a catalyst suitable for the multiphase oxidation of bio‐sourced alcohols. The sample was *ad‐hoc* synthesized by adapting a procedure recently reported by us,^[14]^ in which (NH_4_)MoO_4_ and microcrystalline cellulose (lot # MKBN6866 V), both supplied by Merck, were used as a metal and carbon precursors, respectively. ICP analyses proved that Mo‐loading was 6.1 wt %. It should be noted here that the control of the metal content on the catalyst was challenging because it depended on the nature of the cellulose. It is well‐know that cellulose undergoes depolymerization during its heating; particularly, in a N_2_ atmosphere and above 350 °C, a substantial cleavage of glycosidic bonds occurs with the release of levo‐glucosan and the formation of a charred residue. However, the extent of this degradation process and the corresponding weight loss of the starting biopolymer may change according to its crystallinity.[^30^, ^31^] In this work, the used cellulose proved an excellent choice to ensure a reproducible preparation and to confer hydrophobicity to the final carbon support: the resulting Mo/C sample was able to segregate in a non‐aqueous phase as described above for Ru/C. Details on the synthesis of Mo/C are provided in the experimental section, while its comprehensive characterization is discussed later on.

### Catalysts Characterization

A comprehensive characterization study of the fresh and the used (spent) Mo/C catalyst, before and after the catalytic tests, respectively, was conducted by employing multiple techniques such as TEM, XRD, XPS (Table 1), and N_2_‐physisorption. Particularly, if not otherwise specified, data for the spent sample were gathered after its use for the oxidation of BnOH under the conditions of entry 12, Table 2 (150 °C, 20 bar, 24 h; refer to the catalytic activity section).

**Table 1:** Binding energy values (in eV) and surface chemical composition (at. %) of the constituent element of the Mo catalyst (fresh and spent).

Catalyst	C 1s	O 1s	Mo 3d5/2	C (%)	O (%)	N (%)	Mo (%)	Mo wt %^[a]^	Mo at %^[a]^
Mo/C fresh	284.8 (84 %)	530.9 (42)	229.7 (3)	90.27	8.54	–	1.19	3.53	0.46
	286.1 (10 %)	532.3 (30)	231.1 (6)						
	287.3 (4 %)	533.7 (28)	232.9 (91)						
	288.8 (2 %)								
Mo/C spent	284.8 (80 %)	531.2 (20)	232.6 (100)	86.87	11.45	1.61	0.07	2.09	0.27
	286.2 (11 %)	532.1 (39)							
	287.4 (5 %)	533.6 (41)							
	288.9 (4 %)								

[a] Calculated via EDX.

**Table 2:** The catalytic oxidation of benzyl alcohol in water and in the **MP1–2** systems.

Entry	Reaction medium	Cat.	T/p/t (°C, bar, h)	Conversion (%)	Selectivity (%)	
					PhCHO	PhCO_2_H
1	H_2_O	Ru/C	130, 20, 2	60	88	12
2			130, 20, 4	>99	14	86
3			130, 20, 12	>99	–	>99
4		Mo/C	130, 20, 24	60	>99	–
5			150, 20, 24	>99	10	90
6	**MP1**	Ru/C	130, 20, 4	>99	28	72
7		Mo/C	130, 20, 24	40	>99	–
8			150, 20, 24	98	5	95
9	**MP2**	Ru/C	130, 20, 2	50	>99	–
10			130, 20, 4	>99	>99	–
11		Mo/C	130, 20, 24	45	>99	–
12			150, 20, 24	90	>99	–

#### XRD

**Figure 2:**
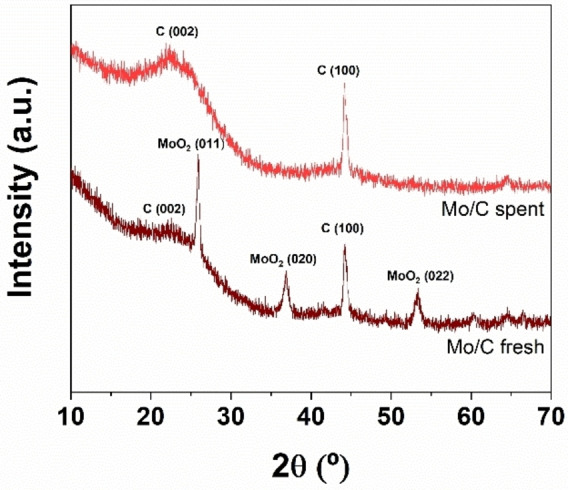
XRD patterns of Mo/C catalyst before (fresh) and after (spent) the oxidation reaction of BnOH (conditions of entry 12, Table 2: 150 °C, 20 bar, 24 h).

The X‐ray diffraction (XRD) patterns of the Mo/C catalyst are illustrated in Figure 2. Both the fresh and the used sample displayed two distinctive peaks at around 25° and 43° which were attributed to the (002) crystallographic plane of the parallel arrangement of graphene‐like layers and the (100) crystallographic plane of graphitic carbon within a honeycomb network, respectively. The broad profile at ca 25° primarily stemmed from the amorphous nature of the samples.

The XRD diffractogram of the Mo/C fresh system also showed well‐defined peaks around 26°, 35°, and 51°, likely indicating the presence of MoO_2_ species in the bulk material. However, these signals were absent in the spent catalyst, possibly due to a reduced metal concentration in this sample.

#### XPS

**Figure 3:**
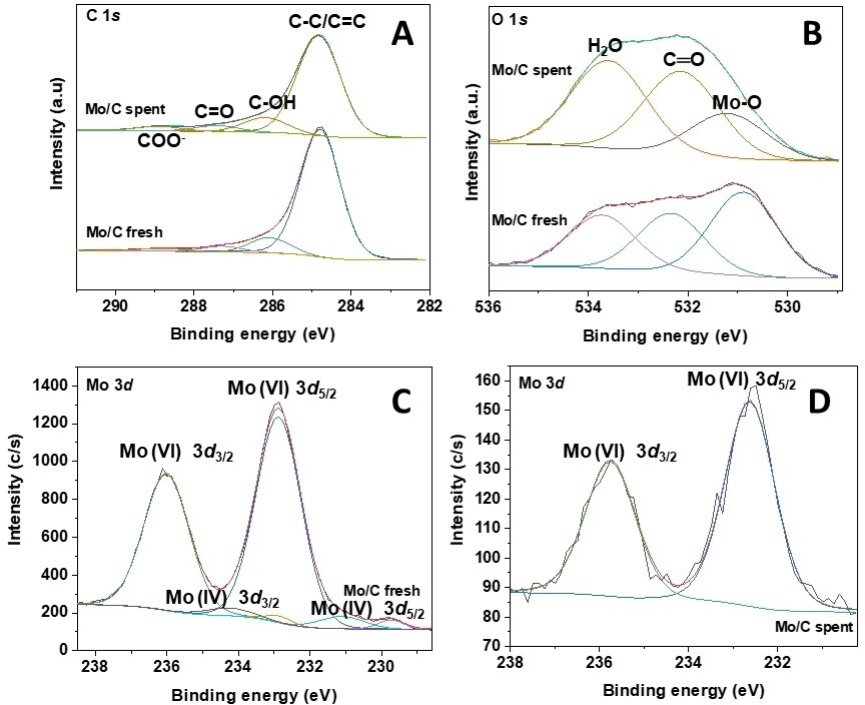
High resolution C 1*s*, O 1*s*, Mo 3*d* core level spectra of Mo/C catalyst before (fresh) and after (spent) the oxidation reaction of BnOH (conditions of entry 12, Table 2:150 °C, 20 bar, 24 h).

The high‐resolution C 1*s*, O 1*s*, and Mo 3*d* core level XP‐spectra are presented in Figure 3, with binding energy values (in eV) and surface chemical composition (in at %) detailed in Table 1. The C 1*s* core level spectrum of the fresh catalyst was deconvoluted into four contributions at 284.8, 286.1, 287.3, and 288.8 eV, attributed to graphitic and adventitious carbon, C−OH and C−O−C bonds, C=O bonds, and carboxylic groups, respectively. The C 1*s* spectrum showed minimal modification in the used sample, presenting four contributions at similar binding energies, albeit with slightly different relative intensities. Additionally, the O 1*s* core level spectra were deconvoluted into three contributions at 530.9, 532.3, and 533.7 eV for the fresh sample and at 531.2, 532.1, and 533.6 eV for the spent catalyst. Although the values were similar, significant differences were noticed in the relative intensities. For instance, contributions at lower binding energy (530.9–531.2 eV), assigned to lattice oxygen of molybdenum oxide, exhibited lower intensity in the spent catalyst. This was consistent with the loss (leaching) of metal during the use of the Mo/C sample. The high‐resolution Mo 3*d* spectrum for the fresh catalyst displayed three doublets Mo 3*d*_5/2_‐Mo 3*d*_3/2_ at 229.7–232.9 eV, 231.1–234.2 eV, and 232.9–236.0 eV. The predominant doublet was assigned to Mo(VI) as MoO_3_, while those at lower binding energy corresponded to reduced Mo species, primarily Mo(IV). Apparently, XRD and XPS measurements indicated the prevalent presence of Mo (IV) species in the bulk material, and of nearly fully oxidized Mo(VI) on the surface of the sample, respectively. A single doublet Mo 3*d*_5/2_‐Mo 3*d*_3/2_ at 232.6–235.8 eV, coherent with the presence of Mo(VI), was observed also in the case of the spent catalyst. However, the analysis of the surface chemical composition of Table 1 revealed that the Mo content decreased significantly from 1.19 % to 0.07 % in the fresh and spent sample, respectively, thereby indicating a substantial Mo leaching after the use of the catalyst. This was further confirmed by the ICP‐MS analyses which determined a metal concentration of 1.5 wt % in the used Mo/C (Table 3), equal approximately to only 25 % of the initial Mo concentration (6.1 wt %) in the fresh specimen.

**Table 3:** ICP‐MS analysis of the **MP2** system and the catalysts after the recycle operations.

Entry	Sample	Mo		Ru
		(μg/mL)^[a]^	(wt %)^[b]^	(μg/mL)^[a]^
1	Water	0.04	–	0.05
2	Isooctane	0.06	–	0.04
3	Ionic liquid	1775	–	15
4	Spent catalyst	–	1.5	–

[a] Metal concentration (μg/mL) in each of the liquid components of the **MP2** system. [b] Content of Mo in the spent catalyst recovered after recycles of Figure 8.

XPS also revealed the presence of a not negligible amount of nitrogen (1.61 %) on the spent sample, which was more than likely related to the adsorption of the ionic liquid ([CH_3_(CH_2_)_6_CH_2_]_3_N(Cl)CH_3_) in the **MP2** system) on the catalyst surface. Apparently, the IL strongly interacted with the carbon support so that it was not removed in the post‐reaction treatment, even after full rinsing by organic solvent during filtration. By contrast, the fresh sample was *N*‐free thereby confirming the full thermal degradation of the precursor (NH_4_)_2_MoO_4_ during the catalyst preparation.

#### HRTEM

**Figure 4:**
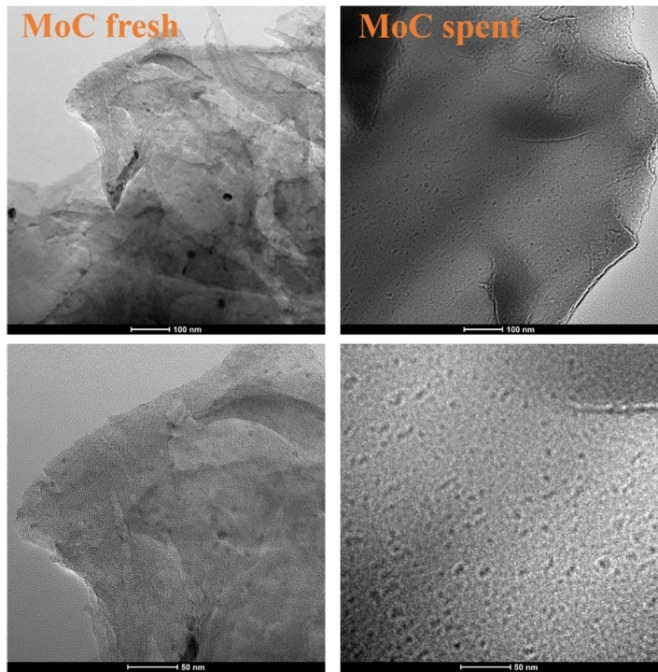
HRTEM images of the fresh and spent catalyst.

**Figure 5:**
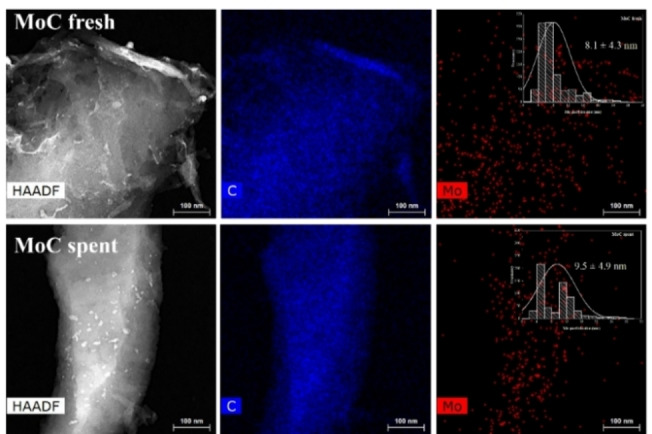
STEM and EDX images of the fresh and spent catalyst, and Mo particle size distribution.

HRTEM micrographs revealed the laminar structure of the carbonaceous support, in the fresh and used catalysts (Figure 4). In both cases, molybdenum nanoparticles were uniformly dispersed within this carbonaceous structure. In the fresh sample, Mo nanoparticles presented an average diameter of 8.1±4.3 nm, with most falling within the range of 4–8 nm. Conversely, the spent catalyst exhibited a bimodal size distribution with most metal particles ranging between 4–6 nm and 10–14 nm (average diameter: 9.5±4.9 nm). Such results suggested a partial sintering of Mo nanoparticles under the conditions employed for the oxidation reaction. Mapping analysis confirmed both a high dispersion and a uniform distribution of the metal (Figure 5). Table 1 presents the atomic concentration percentage and total weight percentage calculated via Energy Dispersive X‐ray Spectroscopy (EDX).

#### N_2_‐Physisorption

**Figure 6:**
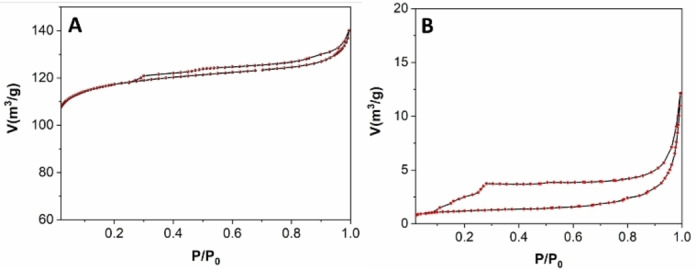
N_2_‐physisorption isotherms of Mo/C catalyst before (A, fresh) and after (B, spent) the oxidation reaction of BnOH (conditions of entry 12, Table 2:150 °C, 20 bar, 24 h).

The fresh Mo/C exhibited a type IV isotherm and type II adsorption hysteresis, indicative of the development of mesoporous materials and the existence of disordered networks (Figure 6). The corresponding measures of the textural properties including surface area, pore diameter, and pore volume, provided values of 520 m^2^/g, 4.3 nm, and 0.2 m^3^/g, respectively. Compared to these features, the textural properties of the spent sample underwent a drastic change, as shown, for example, by the reduction of the surface area down to 4.3 m^2^/g as well as a decrease of the pore volume (0.01 m^3^/g) and a slight increase in mean pore size (6.7 nm). This was attributed to the experimental conditions used for the oxidation tests which resulted in the breakdown of the carbonaceous architecture and the occurrence of pore occlusion phenomena due to the adsorption of organic entities, most likely the ionic liquid, on the catalyst support.

Despite these significant alterations, Mo/C was a recyclable catalyst when used under multiphase conditions (**MP2** system): its catalytic performance did not show any appreciable change even after five subsequent reuses (see later in the catalysts recycling section).

### Catalytic Activity

*The oxidation of benzyl alcohol*. Benzyl alcohol (BnOH) was chosen as a model substrate to begin the investigation. The partial oxidation of BnOH to benzaldehyde was the target of the study. This is a highly desirable reaction in the design of biomass valorisation strategies, not only for BnOH is a representative bio‐based molecule but also for the intrinsic value of benzaldehyde as a versatile intermediate and a flavouring agent.^[14]^ Oxidation tests were carried out in a batch mode using a stainless‐steel autoclave. In the presence of the catalysts above described (5 wt % Ru/C or 6.1 wt % Mo/C; 100 mg each, respectively), three different reaction environments were explored. These were comprised of: i) water (5 mL), ii) a liquid biphase of water and isooctane (5 mL each, system **MP1**), and iii) a liquid triphase of water (5 mL), isooctane (5 mL), and methyltrioctyl ammonium chloride (CH_3_(CH_2_)_6_CH_2_]_3_N(Cl)CH_3_), 500 mg) (system **MP2**). The substrate, BnOH (2 mmol) was dissolved in water according to its solubility limit (3.50 g/100 mL at 20 °C,^[32]^). The resulting solution was 0.4 m. Air was chosen throughout as an oxidant, either in water or in multiphase, to improve the safety and sustainability of the protocol. Further details on the reaction setup are available in the experimental section.

A variety of experiments were carried out by varying the temperature and the pressure in the range of 110–150 °C and 10–50 bar (air), respectively, according to both a literature inspection on the oxidation of BnOH and previous results from our group.^[14]^ The most representative results of this parametric analysis are shown in Table 2 which refers to reactions run at 130–150 °C and at a constant pressure of 20 bar, over a period of time of 2–24 h. Other details of this study are described in the Supporting Information section (Figures S1, S2, and S3, Supporting Information).

All reactions were carried out using an aqueous solution of BnOH (0.4 m, 5 mL) in the presence of Ru/C or Mo/C (100 mg, respectively), and air as an oxidant. **MP1**: isooctane (5 mL) was present. **MP2**: isooctane (5 mL) and methyltrioctyl ammonium chloride (CH_3_(CH_2_)_6_CH_2_]_3_N(Cl)CH_3_); 500 mg) were present. Conversion and products distribution were determined by GC and GC/MS.

In *water*, the Ru‐catalyzed oxidation of BnOH always produced both benzaldehyde and benzoic acid. Even at a moderate conversion (60 %, reached at 130 °C after 2 h), a not negligible amount of the acid was observed (12 %, entry 1). This progressively increased to 86 and 99 % for reactions prolonged up to 4 and 12 h, respectively (entries 2 and 3). Additional tests confirmed that any decrease of T and p was not beneficial because of its adverse effects on the energetics of the reaction and the solubility of the oxidant (O_2_) in the reaction mixture.[^33^, ^34^] Further details are shown in Figure S1, Supporting Information. Mo/C was far less active than Ru/C, though it was apparently more selective. The aldehyde was achieved as an exclusive product (>99 %) at 130 °C, but the conversion did not exceed 60 % after 24 h (entry 4). Increasing the temperature to 150 °C allowed a quantitative reaction; however, it brought about an abrupt drop of the aldehyde selectivity and made benzoic acid becoming the predominant derivative (90 %, entry 5; see also Figure S3A, Supporting Information).

In the **MP1‐2** systems, the relative proportions of each phase were calibrated from our previous works to obtain a perfect separation of the liquid layers in the reactor with water on the bottom, isooctane on top, and the ionic liquid (when present) in the middle. Moreover, even more interesting was that both the C‐supported catalysts appeared perfectly confined in isooctane or alternatively, in the IL phase (Scheme 1; see also Figure S4, Supporting Information). This (confinement) phenomenon was known and expected for Ru/C,[^15^, ^16^] but it could not be taken for granted for other systems, particularly for the Mo/C sample investigated in this work. Further considerations on the reasons for this behaviour are given below in this section.

In the ***MP1** (water/isooctane) system*, a satisfactorily benzaldehyde selectivity was obtained only at a moderate conversion (40 %). A deep oxidation to benzoic acid was prevalent otherwise. The oxidation of BnOH was generally slower in **MP1** because both Ru/C and Mo/C, confined in isooctane, acted at the water‐organic interphase, compared to water where the catalysts were directly suspended in the reactants solution. At 130 °C, this was confirmed by the larger amount of PhCHO observed with Ru (2 h: 28 % vs 14 %; cfr. entries 2 and 6), and the lower conversion reached with Mo (24 h: 40 % vs 60 %; cfr. entries 4 and 7). The result/behaviour was consistent to previous studies on the kinetics of multiphase‐assisted transformations.[^14^, ^15^, ^16^, ^17^] The Mo‐catalyzed reaction was almost quantitative (98 %) only at 150 °C, but it mostly proceeded with the overoxidation of BnOH to benzoic acid (95 %) and in no way could the aldehyde be achieved as an exclusive product (entry 8).

In the ***MP2** (water/isooctane/ionic liquid) system*, an outstanding control of the products distribution was possible, particularly the reaction took place only through the partial oxidation of BnOH to benzaldehyde, which was obtained with full selectivity (>99 %) at a conversion of 90–99 % (entries 9–12; see also Figures S2 and S3B, Supporting Information). Experimental conditions were not altered with respect to those used for reactions in water or in the **MP1** system and once again, confirmed the superior performance of Ru/C which was active at 130 °C in 2–4 h with respect to Mo/C whose reactions required both a higher T and a longer time of 150 °C and 24 h, respectively. In the **MP2** system, the catalyst was confined in the ionic liquid phase, while the reaction occurred in the water solution. The role of isooctane, apparently inconsequential, was necessary to achieve both phase separation and catalyst segregation. This was extensively described in different works by our group.[^15^, ^16^, ^18^]

Several previous investigations demonstrated that the catalyst confinement in both organic media and in ionic liquids, was observed for a variety of C‐supported metals (Pd/C, Pt/C, and Ru/C) and in all cases, it significantly impacted their catalytic performance.[^15^, ^22^, ^35^] This phenomenon is far from being understood because of the complexity of interactions occurring in multiphase systems,^[36]^ but some aspects deserve attention. An in‐depth characterization study of a variety of carbons led to conclude that only some samples could segregate in apolar liquids, specifically those materials combining a low surface acidity (due to carboxylic, phenolic, and lactonic groups) to the presence of Na‐based impurities (0.1– 0.2 %) on the carbon itself. The properties of the supports used in this study for Ru and Mo and specifically, the cellulose‐derived material designed and used to disperse Mo (Figure 2), apparently fulfilled these requisites, and made the investigated catalysts suitable to the confinement in isooctane. Interestingly, the tuning of the hydrophobic/hydrophilic balance was reported by acid pretreatments of a variety of carbons as carbon nanotubes (CNTs), activated carbons (AC), and F‐doped carbons used for the synthesis of Pd‐, Ni‐ and Au‐based catalysts.[^37^, ^38^]

On the other hand, different literature works hypothesized that the embodiment phenomena of metal catalysts dispersed on carbons in IL media was associated to the occurrence of strong polar interactions between the dense and viscous liquids and (mostly) the surface functionalities of the catalysts support.^[29]^ The IL acted concurrently as a catalyst‐philic phase and as an interfacial boundary layer, which mediated the migration (adsorption/desorption) of the liquid/gaseous reagents and products to and from the catalyst, respectively, therefore impacting both conversion and products distribution of the reactions. Whatever the reasons for the effect(s) of the ILs, the catalysts segregation allowed an excellent benzaldehyde selectivity and was advantageous for the separation of the liquid products without resorting to filtration and/or centrifugation. Both these operations are costly and time‐consuming especially for C‐supported systems.[^39^, ^40^]

#### Catalysts Recycling

**Figure 7:**
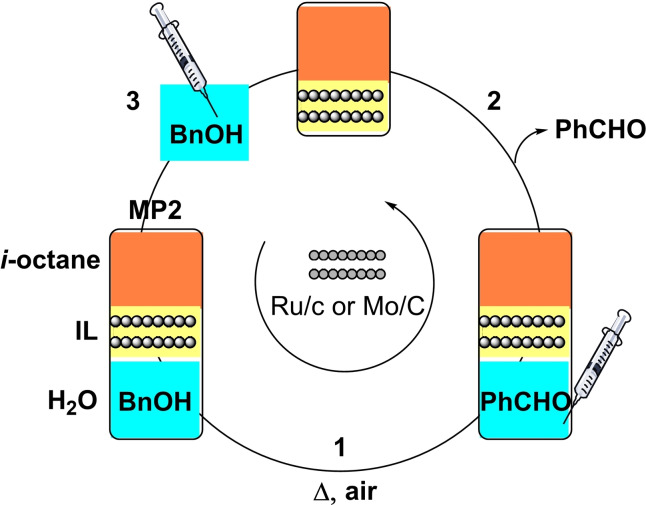
Semicontinuous design for the in‐situ recycling of the catalyst.

The findings of Table 2 demonstrated that Ru/C displayed a higher oxidation activity implying a lower energy demand compared to the reactions catalyzed by Mo. The latter, however, was approximately 340 times less costly than Ru,^[41]^ and more sustainable since not at risk of depletion like Ru. These aspects are critical in any liquid‐phase reaction where a metal‐based catalyst may cost up to one third of the total cost of the process, and its reuse is imperative.^[42]^ In this context, the potential of the **MP2** system was explored to design additional experiments aimed to an *in‐situ* recycle of the catalyst in the ionic liquid phase. The semicontinuous procedure schematized in Figure 7 (steps 1–3) was used.

**Figure 8:**
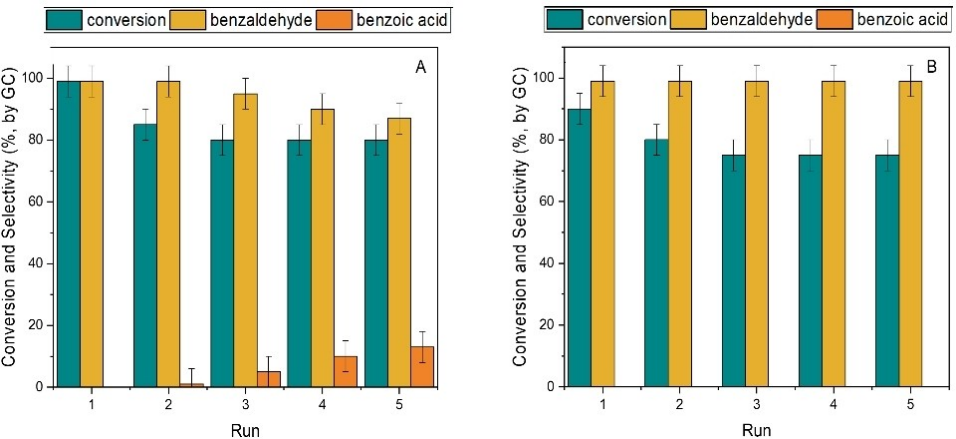
Semicontinuous recycle of the catalyst during the selective oxidation of BnOH to benzaldehyde carried out in the MP2 system. Conditions for each run: aqueous solution of BnOH (0.4 m, 5 mL); isooctane (5 mL), methyltrioctyl ammonium chloride (CH_3_(CH_2_)_6_CH_2_]_3_N(Cl)CH_3_); 500 mg), 20 bar (air). A): Ru/C (100 mg) as the catalyst, 130 °C, 4 h; B) Mo/C (100 mg) as the catalyst, 150 °C, 24 h.

A first reaction was carried out under the conditions of entry 10 or entry 12 of Table 2, using Ru/C or Mo/C, respectively (step 1). After the experiment was complete, the aqueous phase (containing the reaction products) was withdrawn using a syringe (step 2) and replaced with an equal volume of a fresh aqueous solution of BnOH (5 mL, 0.4 m) (step 3). Thereafter, a second oxidation was run. The overall sequence was repeated for five subsequent reactions. Results are reported in Figures 8A,B which refer to the recycle of Ru/C and Mo/C, respectively.

The conversion dropped from 99 % to ≈80 % in the case of Ru/C, and from 90 % to ca 75 % for Mo/C, after the first run. Thereafter, from the second reaction on, it (conversion) remained substantially steady. A similar behaviour was previously noticed by us for other MP‐assisted processes,[^17^, ^22^] and it was explained by the help of the visual inspection of such systems during the recycle operations. At the start of the first reaction, the catalyst appeared dispersed throughout the multiphase, *i. e*. partitioned in the three liquid components (H_2_O, isooctane, IL). This meant that either Ru/C or Mo/C were partially available for the direct adsorption of the reactants in the aqueous phase, thereby favouring the kinetics of the oxidation. Upon stirring the multiphase, however, the catalyst progressively moved in the IL layer where it appeared fully segregated from the first recycle on. Under such conditions, the reaction was slower since it occurred only at the phase boundary. Additional experiments – not reported in Figure 8 – were also carried out even after the fifth use, by further recycling twice the IL‐confined Mo/C, but at a higher temperature of 160 °C. The conversion of these two reactions was not only steady, but it reached the same high value (90 %) observed in the first oxidation test.

Figure 8 also indicated that the selectivity towards benzaldehyde was always ≥99 % in the case of Mo/C, while it progressively decreased to 88 % for the Ru‐catalyzed reactions due to the formation of benzoic acid up to 12 % in the last recycle. This apparent drop in the catalyst performance (selectivity) was no longer investigated. However, among plausible causes, it was hypothesized that the prolonged exposure of Ru nanoparticles to both oxygen and reaction intermediates or by‐products accumulated on their surface across multiple cycles, did play a role in modifying the adsorption mechanism and activation of reactants over Ru/C. This aspect will be the object of future studies.

#### ICP‐MS Analyses and Sheldon Tests

ICP‐MS analyses were performed to shed light on the reliability of the catalyst recycle. Each liquid component of the **MP2** system and the spent catalysts were examined at the end of the experiments of Figure 8. Results are reported in Table 3. (Further details are in the Supporting Information sections).

The presence of both metals, Ru and Mo, in the aqueous solution and in the isooctane was negligible (40—60 ppb, entries 1–2). In the ionic liquid phase, the leaching of Ru was very low (ca 0.15 % of the total metal amount in the starting catalyst) and consistent with the behaviour of the same Ru/C sample in a variety of previously explored reducing/oxidizing conditions,[^16^, ^22^] while the loss of Mo/C was remarkable (entry 3). The amounts of Mo found in IL phase and Mo loading of the spent catalyst were, however, incongruent with the overall mass balance of the metal since they accounted for only 16 % and 25 % (total 41 %), respectively, of the metal concentration in the fresh sample (6.1 wt %). The reasons for this result were not yet clarified, but a hypothesis was formulated based on the presence of nitrogen (1.61 %, Table 1, XPS analyses) on the surface of the used catalyst. This was associated to the adsorption of organic moieties coming from the ionic liquid in the **MP2** system. It was reasonable to assume that this adsorption/deposition on the spent catalyst brought about an increase of its content of carbon and a decrease of its Mo loading, respectively. The adsorption of the IL as such or of species originated from it, was further substantiated by the occlusion of the catalyst pores detected by the N_2_‐physisorption measures (Figure 6).

Sheldon tests were designed to assay the activity of metals leached in the IL phase and compared to a blank multiphase reaction performed without any added catalyst. (Further details are in Table S1, Supporting Information). The corresponding conversion was 21 % and 14 % for the Sheldon test after using Ru and Mo, and 6 % in the blank test, respectively. Notwithstanding its almost insignificant concentration, dissolved Ru displayed a significant activity, but this homogeneous form appeared less selective than the supported metal as suggested by the increasing amount of benzoic acid observed at high conversion, during the catalyst recycles (Figure 8A). By contrast, the comparably higher amount of Mo soluble in the IL phase (whatever this species was) did not affect the formation of benzaldehyde which was always the exclusive product (Figure 8B). This indirectly demonstrated that the multiphase oxidation predominantly took place over the Mo/C solid, the performance of which, particularly the selectivity, was steered by the adsorption of the ionic liquid on its surface. Overall, all characterization analyses were consistent with a profound alteration of the properties of Mo/C during use and recycles, but the resilience of the catalyst was surprising: its confinement in the ionic liquid layer of the multiphase system not only preserved in full its activity and selectivity for five (and more) subsequent reuses, but it facilitated its practical reusability avoiding any metal contamination of the product or metal loss in water/isooctane phases. Once the reasons for Mo‐leaching in the IL‐phase will be further explored in future studies, and possibly, the Mo/C catalyst optimised, this system will be more thoroughly investigated for its potential under MP‐conditions.

#### Substrate Scope

The ternary **MP2** system was further explored to assess its general applicability to the oxidation of other benzyl‐type and aliphatic alcohols. Five benzyl‐type alcohols including 4‐hydroxy‐, 2‐hydroxy‐, 4‐methoxy‐, 4‐hydroxy‐3‐methoxy‐ and 4‐bromo‐ benzyl alcohol (compounds **1–5**, Table 4) were initially considered. Vanillic alcohol (**4**) belongs to the so called ‘‘vanillin platforms” and is among the most important derivatives of the lignin depolymerization,^[43]^ while for anisyl alcohol (**3**) and hydroxy‐substituted benzyl alcohols (**1** and **2**), albeit still prepared from non‐renewable petrochemical resources, innovative bio‐based approaches have been successfully reported using engineered microbes and inexpensive feedstocks from protein waste hydrolysate.[^44^, ^45^] These alcohols and their aldehydes find relevant applications in the cosmetics, perfume and food industries. To the best of our knowledge, 4‐bromobenzyl alcohol (**5**) is not a bio‐based compound, but it represents a good benchmark substrate to compare the oxidation reactivity of the class of alcohols investigated here.

**Table 4:** Oxidation of benzyl‐type alcohols in the **MP2** system using both Ru/C and Mo/C.

Entry	Substrate	Product	Conversion and Selectivity (%)	
			Ru/C	Mo/C
1	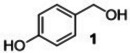	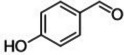	>99, >99	95, >99
2	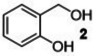	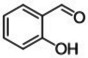	>99, >99	>99, >99
3	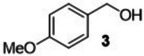	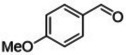	>99, >99	92, 98
4	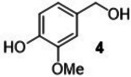	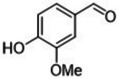	>99, >99	>99, >99
5	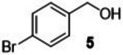	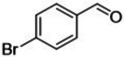	98, 98	76, >99

Reaction conditions were set according to entries 10 and 12 of Table 2 identified for Ru/C and Mo/C, respectively. The results are reported in Table 4.

Both catalysts displayed an exceptional performance with conversion and selectivity to the aldehyde products above 92 % and 98 %, respectively, in all cases except for 4‐bromobenzyl alcohol (**5**) for which a slightly lower conversion was observed for the reaction catalyzed by Mo/C (76 %, entry 5). The same behaviour was noticed and previously reported for compound **3** also during its oxidation over a Mo–N/C system in acetonitrile solvent.^[14]^ Electron‐withdrawing properties of the *p*‐bromide substituent plausibly contributed to this difference. However, regardless of the used substrate, the **MP2** system allowed the perfect segregation of the catalyst in the IL‐phase from the reactants/products in the aqueous solution.

#### Conditions

Aqueous solution of benzyl‐type alcohol (0.4 m, 5 mL), isooctane (5 mL), methyltrioctylammonium chloride ([CH_3_(CH_2_)_6_CH_2_]_3_N(Cl)CH_3_; 500 mg), catalyst (100 mg; Ru/C or Mo/C), 20 bar (air). Temperature and time were set to 130 °C and 4 h, and 150 °C and 24 h for Ru/C and Mo/C, respectively.

*n*‐Butyl alcohol (*n*‐BuOH) was chosen as a model substrate for water‐soluble primary aliphatic alcohols. A preliminary screening highlighted that conditions used for benzyl alcohols were not suitable for *n*‐BuOH. A parametric analysis of the oxidation of *n*‐BuOH was then carried out by varying the amount of the catalyst, the temperature, the air pressure, and the reaction time. Details of this study are shown in Figure S5A−F, Supporting Information. Table 5 summarizes the most representative results.

**Table 5:** The oxidation of *n*‐BuOH utilizing Ru/C and Mo/C systems in water and in the **MP2** system.

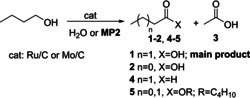									
Entry	Reaction medium	T/p/t (°C, bar, h)	Cat.	Conversion (%)	Products distribution (%)				
					1	2	3	4	5
1	H_2_O	150, 50, 6	Ru/C	>99	70	23	6	–	1
2	**MP2**				75	20	3	1	1
3	**MP2**	150, 50, 24	Mo/C	42	36	15	4	15	30

#### Other Conditions

An aqueous solution of *n*‐BuOH (0.5 m, 5 mL), catalyst (50 mg). **MP2** system: isooctane (5 mL), [CH_3_(CH_2_)_6_CH_2_]_3_N(Cl)CH_3_ (500 mg) (entries 2 and 3).

Whatever the reaction environment (H_2_O or **MP2**), at complete conversion, the Ru/C‐catalyzed reaction proceeded with the predominant formation of butyric acid (**1**: 70—75 %) and only trace amounts of butanal (**4**: 1 %) (entries 1–2). The (over)oxidation of *n*‐BuOH occurred along with a competitive process of carbon‐carbon bond cleavage yielding shorter chain acid derivatives as propanoic acid (**2**: 20—23 %) and acetic acid (**3**: 3—6 %). Further considerations on the oxidative C−C bond cleavage of unstrained alcohols are discussed in the Supporting Information. An esterification side‐reaction also took place to a minor extent affording *n*‐butyl esters (**5**: 1 %). The control of the products distribution was even less satisfactory using Mo/C as a catalyst in the **MP2** system. Not only a moderate conversion (42 %) was reached after 24 h, but a much poorer oxidation selectivity was observed: comparable amounts of butyric acid (36 %) and butyl esters (30 %) were obtained as well as of propionic acid and butanal (15 % each) (entry 3).

In the **MP2** system, however, the confinement of Ru/C or Mo/C was still effective in the IL phase, so that the catalyst/product separation was successful. The catalyst recycle – though not explored – was likely possible under such conditions.

#### Comparison to Literature Results

A comparative assessment of the **MP2**‐assisted protocol proposed in this work against a selection of representative methods available in the literature for the selective oxidation of BnOH was finally carried out. Reactions designed in water and water/organic biphase and in the presence of supported Ru or Mo catalysts were considered. Results are summarized in Table 6. Literature procedures generally reported conditions milder than those used in this work, with T not exceeding 90 °C and ambient pressure, but they required both strong hazardous oxidants as oxygen and concentrated H_2_O_2_, and a complex/multistep design of catalysts, co‐catalysts and reactors. In the case of Ru, this meant: i) the fabrication of single atoms (SA) anchored onto a nitrogen‐doped carbon (entry 1) or bimetallic nanoparticles supported on carbon nanotubes (entry 2), and ii) the use of randomly methylated β‐cyclodextrins as co‐catalysts (entry 3) or a superamphiphilic carbon (entry 4) which made the metal active in Pickering emulsions. For Mo, instead, the catalyst active phase was either made to flow through a flat membrane reactor fitted with a hydrophobic polymeric film (entry 6) or made available as a pyridinium exchanged α‐Kegging anion, not suitable for recycle (entry 7). Overall, though inventive and effective, all these methods implied the engineering of costly and environmentally impactful syntheses and the use of expensive starting materials to obtain active metals and suitable supports and reactors.

**Table 6:** Comparison of the **MP2**‐assisted procedure to selected literature methods for the oxidation of BnOH in water and water/organic biphase.

Entry	Cat. (recycle)	Oxidant	Reaction medium	T/p/t (°C/bar/h)	Conv.’n/Sel. (%)	Ref.
1	Ru_1_/NC^[a]^ (yes)	O_2_	Water	90/1/2	99/99	[46]
2	Pt_x_Ru_y_/CNT^[b]^ (none)	O_2_	Water	80/1/3	59/90	[47]
3	Ru/C‐RaMeβ‐CD^[c]^ (yes)	O_2_	Water	75/n.i.^[d]^/24	99/99	[48]
4	Ru/C – sa. C^[e]^ (none)	H_2_O_2_	Water/toluene	90/1/2.5	94/94	[49]
5	Ru/C (yes)	Air	**MP2**	130/20/4	99/99	This work
6	(NH_4_)_6_Mo_7_O_24_ 4H_2_O^[f]^ (none)	H_2_O_2_	Water/alcohol	60/n.i. ^[d]^/4	48/98	[50]
7	Py_3_PMo_12_O_40_^[g]^ (none)	H_2_O_2_	Water/organic	70/1/3	n.i.^[d]^/85	[51]
8	Mo/C (yes)	Air	**MP2**	150/20/24	90/99	This work

[a] Ru single atoms (SA) anchored onto a geometrically deformed nitrogen‐doped carbon (Ru1/NC) support. [b] Bimetallic nanoparticles supported on carbon nanotubes (CNT). [c] 10 % Ru/C with randomly methylated β‐cyclodextrins. [d] n.i.: not indicated. [e] 5 % Ru/C in combination with a superamphiphilic carbon (s. a. C). [f] Ammonium molybdate in a flat membrane reactor. [g] A pyridinium substituted heteropolyacid used in combination with ionic liquids.

By contrast, the **MP2**‐based procedure was conceived for being easily accessible via simple and safe combinations of catalyst/conditions for oxidation reactions in water. This was achieved using air as an oxidant, cheap commercially available compounds ([CH_3_(CH_2_)_6_CH_2_]_3_N(Cl)CH_3_, isooctane), and catalysts which were sourced from conventional suppliers (Ru/C from Merck) or prepared by a straightforward synthesis (Mo/C) still from commercial inexpensive precursors (ammonium molybdate and cellulose). The multiphase protocol did not ensure the catalyst stability at least for Mo/C, but nonetheless it made possible the control of the reaction selectivity and an effective recycle across successive runs. Two more considerations should be finally placed based on the CHEM21 toolkit for green practices in chemical synthesis:^[52]^ i) a recent evaluation of ionic liquids through the metrics of this standard, ranked onium salts as tetralkyl substituted ammonium cations and chloride anion as green compounds/media for their positive technoeconomic impact; ii) processes at T≥150 °C generally raise concerns for the energy costs. This aspect, however, appeared mitigated, if not offset, for **MP2**‐based oxidations catalysed by Mo, an element which is characterized by a price steadier and far lower compared to precious metals.^[53]^

## Conclusions

The proposed study unveils insights into a pivotal process of contemporary chemical reactions as the catalytic oxidation of primary benzyl‐type alcohols. The design of an innovative sustainable method is described where the potential of multiphase systems is integrated with the use of air as a green and safe oxidant, and carbon supported catalysts based on environmentally desirable metals, particularly the non‐noble Mo. In the presence of an *ad‐hoc* prepared Mo/C or a commercial Ru/C catalysts, the comparison of different reaction environments proves that a specially designed liquid triphase system (**MP2**: H_2_O/*i‐*octane/[CH_3_(CH_2_)_6_CH_2_]_3_N(Cl)CH_3_) is an extraordinary option to control the products distribution of the oxidation of benzyl alcohol. The selectivity towards the formation of the partially oxidized derivative, benzaldehyde, can be enhanced up to 99 % at complete conversion. The general viability of the multiphase protocol has been confirmed for a small, but representative library of benzyl alcohols (five examples): substrates bearing *p*‐ and *o*‐substituents, either EWG or EDG groups, have been quantitatively transformed into the corresponding aldehydes. Crucial to these results is the role of the ionic liquid component ([CH_3_(CH_2_)_6_CH_2_]_3_N(Cl)CH_3_) of the multiphase system that acts as a catalyst‐philic phase able to segregate both Mo/C and Ru/C and keep them separated from water‐soluble reactants and products. This confinement phenomenon, mostly due to the properties of the carbon supports of the catalysts and their adsorption/interactions with the IL, makes the oxidation proceeds as an interfacial process which is not only critical to the selectivity control, but it also allows the recovery of products from an aqueous medium, free from metal contaminations, and an *in‐situ* semicontinuous recycle of both catalysts. The behaviour of the two catalytic systems, however, has been different from each other upon subsequent reuses. Ru/C is comparably more active and stable over time, while Mo/C undergoes both a leaching of the active metal in the ionic liquid phase and a significant alteration of its morphological properties and carbonaceous architecture. Notwithstanding this, Mo/C is extremely resilient during recycles allowing a steady conversion from one run to another and a selectivity (always >99 % to benzaldehyde) even better than that achieved with Ru/C. The reasons of this robustness are not yet clarified: albeit the cellulose‐derived carbon support and/or the procedure used to prepare Mo/C do not preserve its structure under multiphase conditions, they might allow a dynamic leaching/adsorption of the metal in the IL and on the support which confers the peculiar behaviour of the catalyst in the recycle operations. It is agreed that leaching and sintering are detrimental phenomena for a catalyst. However, in the specific case of Mo/C, adverse effects due to alterations of its properties appear strongly mitigated, if not offset at all, by the experimental conditions studied here. The integration of Mo/C in a MP system offer a unique design fulfilling requisites of practical reusability of the catalyst with performance integrity and prevention from metal contamination of the product and/or metal loss, which in the last analysis, are compelling evidence towards improving of the process sustainability.

The **MP2**‐assisted protocol (nor the same reaction in water or water/isooctane) does not prove equally efficient for aliphatic alcohols. As demonstrated for the case of *n*‐butyl alcohol, an overoxidation and an oxidative C−C bond cleavage through a β‐carbon elimination mechanism, take place producing carboxylic acids and shorter chain acid derivatives. Both reactions are interesting, but their competitiveness and modest selectivity makes the procedure less attractive at the present stage. This aspect along with the design of more stable Mo‐based catalysts offer some stimulating challenges that will be the object of future studies in our Labs.

## Experimental Section

### Materials and Equipment

Benzyl alcohols (XC_6_H_4_CH_2_OH; X=H, *p*‐Br, *p*‐OCH_3_, *p*‐OH, *o*‐OH, *m*‐OCH_3_, *p*‐OH), *n*‐butyl alcohol, (NH_4_)MoO_4_, 5 % Ru/C, 2‐propanol, microcrystalline cellulose were commercially available compounds sourced from Sigma‐Aldrich. If not otherwise specified, reagents and solvents were employed without further purification. Compressed air was supplied from SIAD, Italy. GC‐MS (EI, 70 eV) analyses were performed on an HP5‐MS capillary column (L=30 m, Ø=0.32 mm, film=0.25 mm). GC (flame ionization detector; FID) analyses were performed with an Elite‐624 capillary column (L=30 m, Ø=0.32 mm, film=1.8 mm). ^1^H, ^13^C NMR spectra were recorded using a Bruker Advance III HD 400 WB equipped with a 4 mm CP/MAS probe, at 400 and 101 MHz, respectively. Chemical shifts were reported downfield from tetramethyl silane (TMS) and MeOD, DMSO‐d_6_ and CDCl_3_ were used as solvents. Further equipment is described in the catalyst′s characterization section.

#### Synthesis of the Mo/C Catalyst

**Figure 9:**
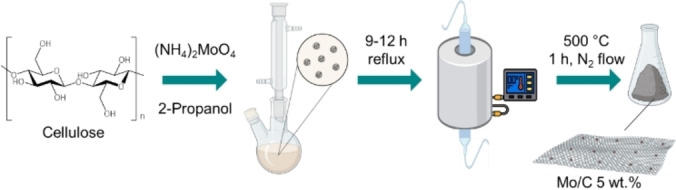
Schematic representation of the two‐step synthetic protocol of the Mo/C catalytic material.

The preparation of the Mo/C sample was accomplished by adjusting a procedure recently reported by our group and based on a two‐step run‐in protocol (Figure 9).^[14]^

(NH_4_)_2_MoO_4_ (1 mmol) was dissolved in 2‐propanol (60 mL) under stirring. The solution was then added with microcrystalline cellulose (5 g) and kept under magnetic stirring for 9 h at the reflux temperature (80 °C). The suspension was filtered and dried at 100 °C overnight, and the recovered solid was subjected to a thermal treatment at 500 °C under a N_2_ flow for 1 h (10 mL/min, heating rate of 5 °C/min). The final sample was ground to powder, stored in a glass vial, and labelled as Mo/C.

#### Catalyst Characterization Analyses

Both fresh and exhausted catalysts used in this work were characterized by a multi‐technique approach. The X‐Ray Diffraction (XRD) analysis, using Cu Kα radiation as the X‐Ray source, was employed to examine the crystalline structure of the samples. This involved monitoring the 2θ values within the range of 10–80° at a rate of 0.08° min^−1^. Textural properties such as surface area, pore volume, and pore size were determined through N_2_ physisorption at ‐196 °C. The samples underwent a 2‐h outgassing at 120 °C before recording adsorption–desorption isotherms at −196 °C. Specific surface areas, pore volumes, and pore size distributions were calculated using the BET method for adsorption isotherms and the Barrett, Joyner, and Halenda (BJH) algorithm. The surface of the fresh and used catalysts were analyzed by X‐ray photoelectron spectroscopy (XPS). The experiments were carried out in an ultrahigh vacuum chamber using a Physical Electronics VersaProbe II Scanning XPS spectrometer equipped with a monochromatic X‐ray Al Kα source (1486.6 eV). The C 1*s* line of adventitious carbon was used for binding energies calibration at 284.8 eV. The accuracy of the binding energy (BE) values was ±0.2 eV. ICP–MS analyses were carried out in an Elan DRC‐e (PerkinElmer SCIEX) spectrometer, to quantify both the metal content of the samples and the metal leaching after the catalysts use. To the scope, fresh and used (post‐reaction) catalysts were subjected to a digestion procedure in the presence of a highly oxidant solution under MW irradiation. Details are reported in the Supporting Information.

The materials morphology and nanoparticle distribution were obtained with a High‐Resolution Transmission Electron Microscopy (TALOS F200x), that also operate in STEM mode (Scanning Transmission Electron Microscopy). The instrument possesses a HAADF detector, working at 200 kV and 200 nA. The mapping images were obtained with an EDX Super‐X system provided with 4 X‐ray detectors and an X‐FEG beam. The molybdenum particle size distribution was performed by Image J software, counting at least 700 particles selected in several different parts of each sample.

#### Catalytic Activity

The oxidation of alcohols was explored using both Ru/C and Mo/C catalysts and air as an oxidant. All reactions were performed in duplicate to verify reproducibility. Unless otherwise stated, conversions, selectivity, and yields (determined by GC‐FID and GC‐MS) differed by less than 5 % from one test to another. The structure of the products was assigned by GC/MS. Multiple consecutive/parallel processes of oxidation (to aldehydes and carboxylic acids), C−C bond cleavage and esterification were observed in this work. Accordingly, the selectivity towards any of the observed products was not defined for a single reaction but calculated with respect to the total amount of the products derived by all the occurring processes. The following expression was used:

$$S_{i}=\left[{mol}_{i}/{con}_{v}\times alcohol\right]\times 100$$

where S_*i*_ is the selectivity (%) for the single compound *i* and mol_*i*_ stands for the total moles of compound *i* (by GC calibration).

Three different reaction environments [i)‐iii)] were examined.

- *Water*. A 15‐mL round‐bottomed glass reactor was charged with a solution of the chosen alcohol (2 mmol) in water (5 mL) and the catalyst (100 mg). The reactor was placed in a stainless‐steel autoclave equipped with two needle valves and a manometer, charged with air at 8–20 bar, and heated under magnetic stirring at the desired temperature for 2–24 h. The autoclave was then cooled to rt, gently purged, and opened. The aqueous phase was syringed out, strained through a microporous filter (0.22 μm) and analyzed by GC and GC/MS.
- *Water/isooctane*. The procedure above described for water was followed with the further addition of isooctane (5 mL) to obtain a biphase liquid system (**MP1**). Interestingly, the catalyst appeared clearly confined within the organic upper phase (isooctane). The reaction and the resulting final mixture were subjected to the same treatment outlined in point i).
- Water/isooctane/[CH_3_(CH_2_)_6_CH_2_]_3_N(Cl)CH_3_. The procedure above described for water was followed with the further addition of isooctane (5 mL) and *N*‐methyl‐*N,N,N*‐trioctylammonium chloride (500 mg) as an ionic liquid to obtain a triphase liquid system (**MP2**). Interestingly, the catalyst appeared clearly confined within the ionic liquid phase (middle layer). The reaction and the resulting final mixture were subjected to the same treatment outlined in point i).

#### Catalysts Recycle

The recycle of the catalysts was investigated when the **MP2** system was used. Once a (first) reaction cycle was complete, the aqueous phase containing the product(s) was syringed out and replaced by an equivalent volume (5 mL) of fresh solution of the reactant alcohol. Then, a second oxidation test was run. The overall sequence was repeated for five subsequent experiments. Both the IL‐phase containing the catalyst and the hydrocarbon were never removed from the reactor.

## Conflict of Interests

There is not any conflict of interest.

1

## Supporting information

As a service to our authors and readers, this journal provides supporting information supplied by the authors. Such materials are peer reviewed and may be re‐organized for online delivery, but are not copy‐edited or typeset. Technical support issues arising from supporting information (other than missing files) should be addressed to the authors.

Supporting Information

## Data Availability

The data that support the findings of this study are available in the supplementary material of this article.
