# Supplementary material for: Selective Multiphase‐Assisted Oxidation of Bio‐Sourced Primary Alcohols over Ru‐ and Mo‐ Carbon Supported Catalysts
Source: ChemSusChem. 2024 Nov 8;18(2):e202400888. doi: 10.1002/cssc.202400888 (PMC11739854; doi:10.1002/cssc.202400888)
Supplement: Supplementary file 1 — Supporting Information [file CSSC-18-e202400888-s001.pdf]

ChemSusChem

Supporting Information

Selective Multiphase-Assisted Oxidation of Bio-Sourced Primary Alcohols over Ru- and Mo- Carbon Supported Catalysts

Chiara Bersani, Daily Rodríguez-Padrón, Daniel Ballesteros, Enrique Rodríguez-Castellón, Alvise Perosa, and Maurizio Selva*

SUPPORTING INFORMATION

Selective Multiphase-Assisted Oxidation of Bio-Sourced Primary Alcohols over Ru- and Mo-Supported Catalysts

Chiara Bersani,[1] Daily Rodríguez-Padrón,[1] Daniel Ballesteros,[2] Enrique Rodríguez-Castellón,[2] Alvise Perosa,[1] Maurizio Selva*[1]

[1] Department of Molecular Sciences and Nanosystems, Università Ca' Foscari Venezia, Via Torino 155, 30172 Venezia Mestre, Italy

[2] Department of Inorganic Chemistry, Facultad de Ciencias, Universidad de Málaga, Campus de Teatinos s/n, 29071 Málaga, Spain

corresponding: selva@unive.it

Table of Contents

Benzyl alcohol oxidation in water and in the MP2 system 2

5 wt.% Ru/C as a catalyst – effects of t, T, and p .... **Errore. Il segnalibro non è definito.**

5 wt.% Mo /C as a catalyst – effects of T .... **Errore. Il segnalibro non è definito.**

Suspension and segregation of Ru/C and Mo/C catalysts in water and in the MP2 system 4

n-BuOH oxidation reaction in water and in the MP2 system - parametrical analysis. 4

XPS Spectra 5

ICP-MS analysis 6

Effects of the reaction time, temperature, and pressure on Ru/C catalysed oxidation of BnOH in water.

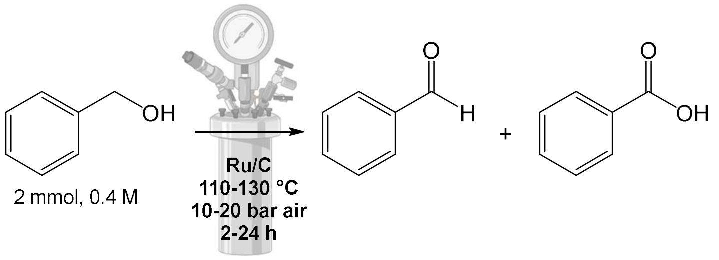

The catalyst used for this study was a 5% Ru/C sample sourced by Aldrich (lot# MKBW5890V). The full characterization of this system for its structural, morphological, and acid properties was reported in previous work by our group.[1,2]

The parametric analysis of the reaction confirmed that in water, any variation of T and p was not beneficial to the selectivity of the reaction.

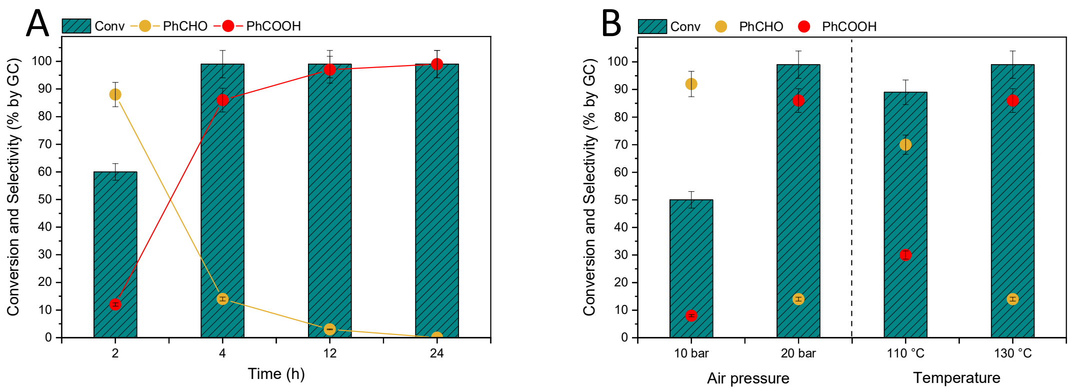

Figure S1. The Ru/C catalysed oxidation of BnOH in water. **A** (left): Effect of the reaction time (from 2 to 24 h) on the reaction conversion and products distribution; **B** (right): Effects of the p from 10 to 20 bar, and T from 110 to 130 °C, on the reaction conversion and products distribution. Other conditions were those of entries 1-3 of Table 2 in the main text [BnOH (2 mmol) in H_2O (5 mL; 0.4 M), 5% Ru/C (100 mg)]. For Figure **S1B**, the reaction time was 4 h.

It should be noted for example that at 130 °C, the conversion did not exceed 50% after 4 h when the pressure was decreased from 20 to 10 bar. The result was consistent with the drop of the oxidant (O_2) available in the reaction mixture as the pressure was reduced.[3,4]

*Effect of the reaction time on the Ru/C catalysed oxidation of BnOH in the **MP2** system*

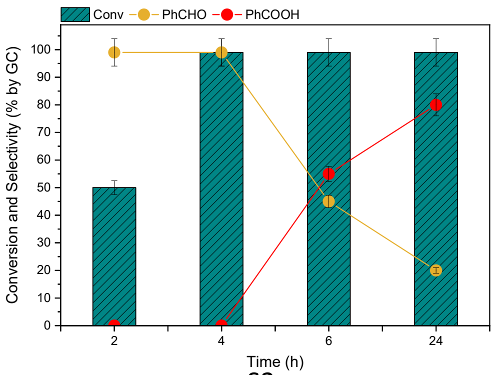

Figure S2. The Ru/C catalysed oxidation of BnOH in the **MP2** system. Effect of the reaction time on the reaction conversion and products distribution. Other conditions were those of entries 9-10 of Table 2 in the main text [BnOH (2 mmol) in H_2O (5 mL; 0.4 M), isooctane (5 mL), [N_{8881}][Cl] (500 mg), 5% Ru/C (100 mg), 130 °C, 20 bar air].

Results demonstrated that in **MP2** system, both the conversion and the benzaldehyde selectivity reached a maximum (>99%, each), after 4 h.

Effects of the temperature on the Mo/C catalysed oxidation of BnOH in both water and multiphase conditions.

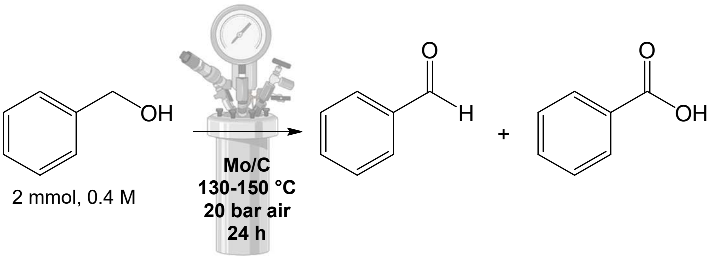

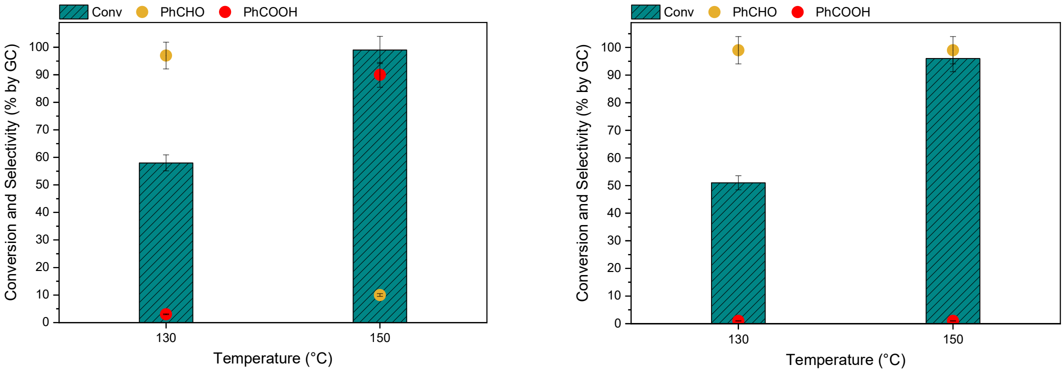

Figure S3. Experiments were carried out in water and in the **MP2** system. The corresponding results are in Figure **A** (left) and **B** (right), respectively. Other conditions were those of entries 4-5 and 11-12 of Table 2 in the main text [BnOH (2 mmol) in H_2O (5 mL; 0.4 M), isooctane (5 mL), [N_{8881}][Cl] (500 mg), Mo/C (100 mg), 20 bar air, 24 h].

Results demonstrated that in **MP2** system, both the conversion and the benzaldehyde selectivity reached a maximum of 90-96% and >99%, respectively.

Suspension in water (left) and confinement in the MP2 system (right) of both Ru/C and Mo/C catalysts studied in this work.

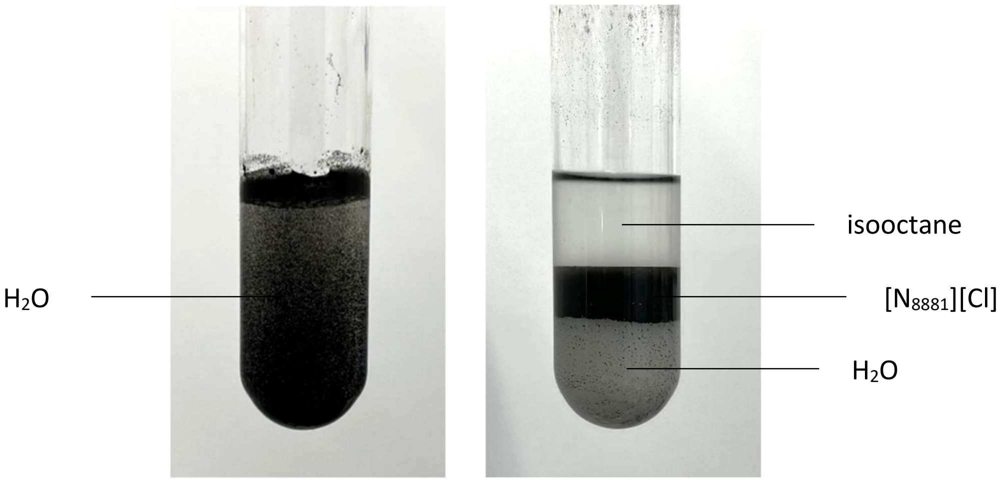

Figure S4. Ru/C and Mo/C in water (left) and in the **MP2** system (right).

n-BuOH oxidation reaction in water and in the MP2 system.

A parametric analysis of the oxidation of n-BuOH was performed with Ru/C by varying: i) the amount of the catalyst, from 15 to 130 mg; ii) the temperature, from 110 to 150 °C; iii) the air pressure, from ambient to 50 bar, and iv) the reaction time, from 2 to 6 h. Experiments were run using an aqueous solution of *n*-BuOH (0.5 M, 5 mL) that was employed as such or in combination with isooctane (5 mL)/([N_{8881}][Cl]) (500 mg) to generate the **MP2** system. Figures S5A-D refer to the reaction carried out in water. Figure S5E refer to the reaction carried out in the **MP2** system.

Additional experiments were performed also by using Mo/C under multiphase conditions (Figure S5F)

Apparently, the best results were achieved in water [150 °C, 50 bar air, 6 h, 5% Ru/C (25 mg)]. 96% conversion and 75% selectivity towards butyric acid were achieved.

The observed C-C bond (oxidative) cleavage of unstrained alcohols is not a new process. Excellent reviews have been reported on this subject.[5,6] Due to the inertness of the Csp^3-Csp^3 σ bond, the cleavage requires activation by transition metals and the predominant pathway for such a process is β-carbon elimination. The metal initially coordinates via the oxygen atom, and this enables the interaction with an adjacent group in β-position which induces the cleavage of the bond. Interestingly, under the conditions of Scheme 2 (Ru/C, H_2O), an additional experiment carried out by replacing *n*-BuOH with butyric acid, showed that the acid was recovered unaltered after 6 h at 150 °C, thereby suggesting that the β-carbon elimination mechanism was exclusive for the alcohol. A strategy based on β-C-C bond breaking coupled to decarboxylation has been recently demonstrated also for carboxylic acids, but it required copper-mediated photoredox catalysis.[7]

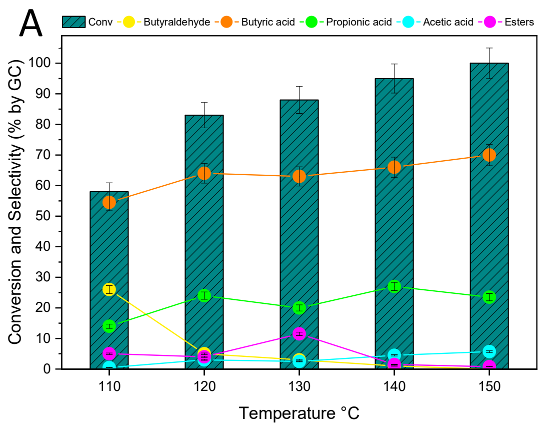

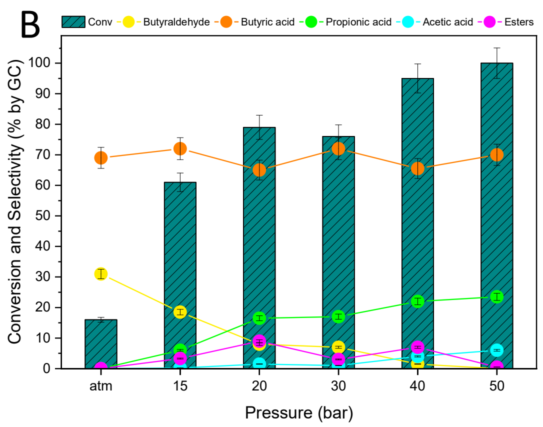

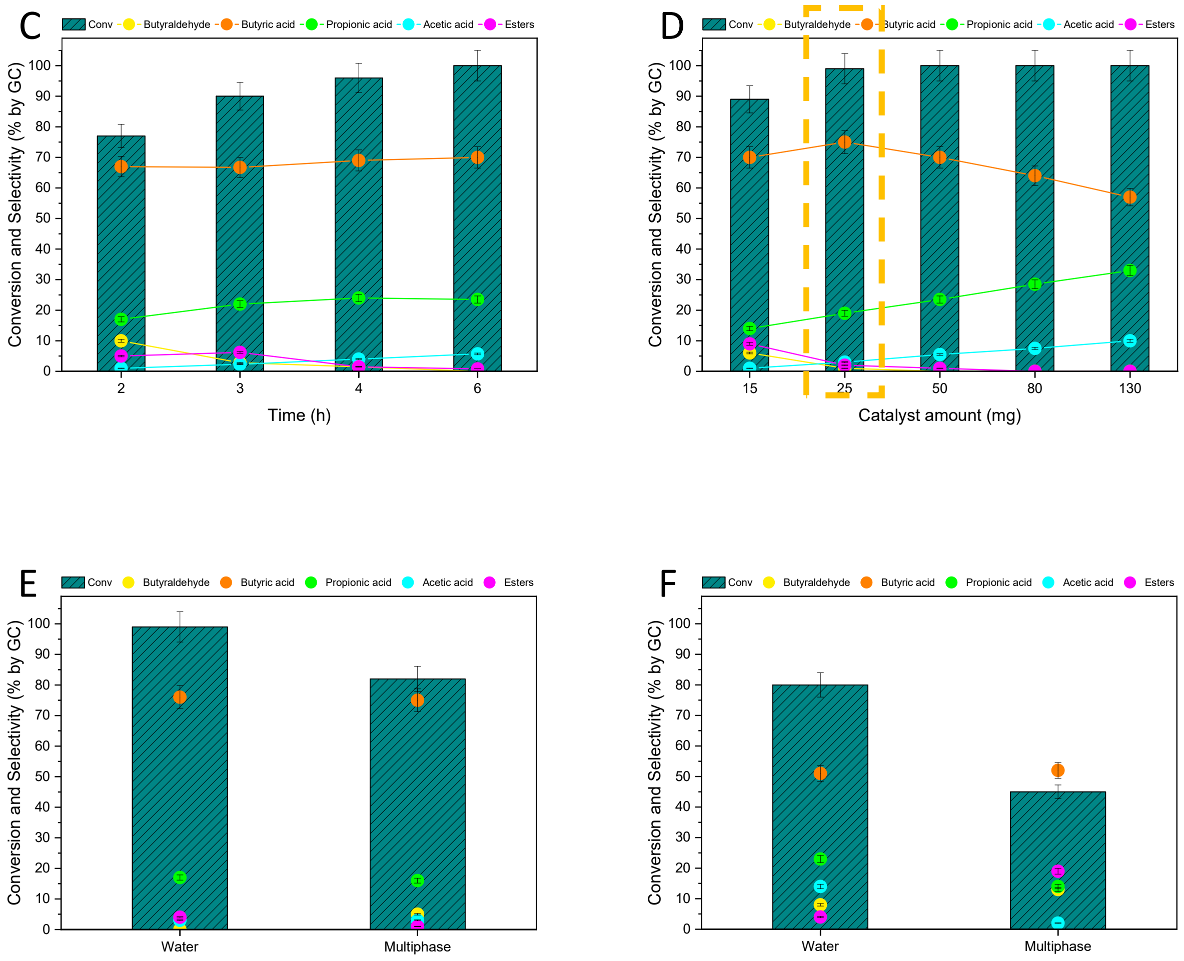

Figure S5. Conditions for oxidation reaction in **water** (Figures **S5A-D**). Figure **S5A** shows the effect of T from 110 to 150 °C [other conditions: 5% Ru/C (50 mg), 50 bar air, 6 h]; Figure **S5B** shows effect of the pressure from ambient to 50 bar [other conditions: 5% Ru/C (50 mg), 150 °C, 6 h]; Figure **S5C** shows the effect of the reaction time from 2 to 6 h [other conditions: 5% Ru/C (50 mg), 150 °C, 50 bar air]; Figure **S5D** shows the effect of the commercial 5 wt.% Ru/C catalyst amount from 15 to 130 mg [other conditions: 150 °C, 50 bar air, 6 h].
Conditions for oxidation reaction in **the MP2 system** (Figures **S5E-F**). Figure **S5E** refers to the reaction carried out at for 6 h at 150 °C, 50 bar air, and using 5% Ru/C (25 mg); Figure **S5F** refers to the reaction carried out at for 24 h, at 150 °C, 50 bar air, and using Mo/C (200 mg).

XPS Spectra

The survey spectra (**Figure S6**) show the presence of C, O and Mo, but in the case of the spent catalyst, the relative intensities of the Mo 3d and Mo 3p signals are very weak, indicating the leaching of Mo.

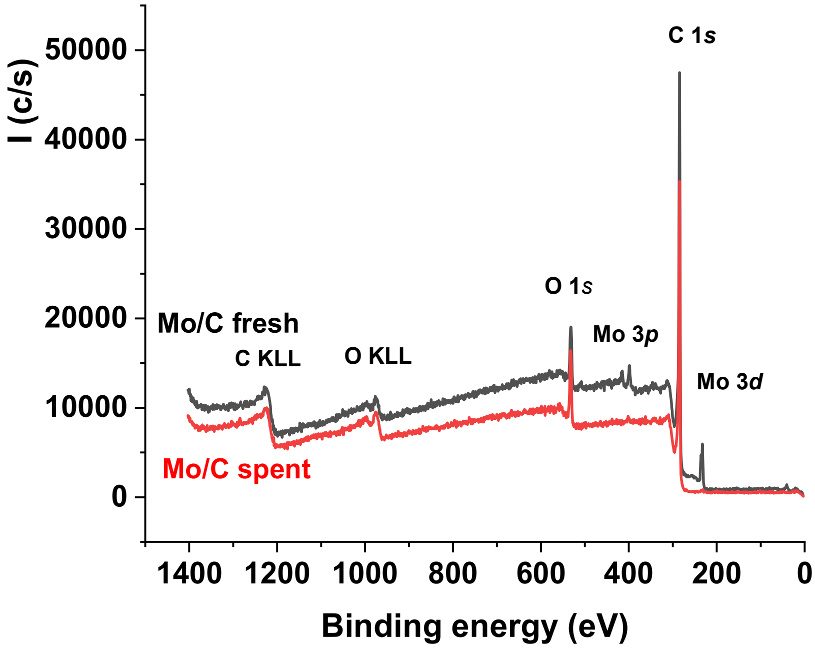

Figure S6. XPS survey spectra for Mo/C fresh and Mo/C spent.

ICP-MS analyses

Analyses were performed on each liquid component of the **MP2** system and on the spent catalysts. At the end of the experiments of Figure 4, water and isooctane were quantitively withdrawn by a syringe from the reactor. The ionic liquid phase was doubly filtrated over a microporous membrane (0.22 µm) to remove the solid catalyst. The three clear liquid phases (H_2O, *i*-octane, and the IL) were then subjected to ICP analyses.

After filtration, the solid catalyst was recovered and dissolved completely in a water solution by combining strong oxidizing conditions and MW heating according to a procedure reported elsewhere.[8]

The Sheldon test

An oxidation experiment was carried out under the conditions of Figure 4 (Ru: 130 °C, 20 bar, 4 h; Mo: 150 °C, 20 bar, 24 h). Thereafter, the solid catalyst suspended in the **MP2** system was filtered on a microporous membrane (0.22 µm), and the aqueous phase containing the product was totally removed and replaced by a fresh aqueous solution of BnOH (0.4 M, 5 mL). A second oxidation run was then started according to the procedure above described in Figure 6. Parallelly, a blank multiphase reaction was performed without any added catalyst. In all cases, the reaction conversion, the selectivity and the products structures were determined/assigned by GC and GC/MS. Results are reported in Table S1

Table S1. Sheldon tests for the oxidation of benzyl alcohol in the **MP2** system.

Entry	Removed catalyst	T/p/t (°C, bar, h)	Conversion (%)	Selectivity (%)[a]
1	**Ru/C**	130/20/4	21%	>99%

2	**Mo/C**	150/20/24	14%	>99%
3	**None (blank)**	150/20/24	6%	>99%

[a] Selectivity to benzaldehyde

The corresponding conversion was 21% and 14% for the Sheldon test after using Ru and Mo, and 6% in the blank test, respectively. At this low-moderate conversion, the selectivity to benzaldehyde was >99% in all cases.

References

[1] A. Bellè, T. Tabanelli, G. Fiorani, A. Perosa, F. Cavani, M. Selva, *ChemSusChem* **2019**, *12*, 3343–3354.
[2] D. Polidoro, A. Perosa, M. Selva, *ChemSusChem* **2022**, *15*, e202201059, DOI 10.1002/cssc.202201059.
[3] A. Pray, C. E. Schweickert, B. H. Minnich, *Ind. Eng. Chem*. **1952**, *44*, 1146-1151
[4] D. Tromans *Ind. Eng. Chem. Res.* **2000,** *39,* 805-812
[5] M. D. R. Lutz, B. Morandi *Chem. Rev.* **2021**, *121*, 300-326
[6] H. Liu, M. Feng, X. Jiang *Chem. Asian J.* **2014**, *9*, 3360 – 3389
[7] R. Li, Y. Dong , S. N. Khan, M. K. Zaman, J. Zhou, P. Miao, L. Hu, Z. Sun *Nat Commun* **2022**, *13*, 7061
[8] A. Bellè, K. Kusada, H. Kitagawa, A. Perosa, L. Castoldi, D. Polidoro, M. Selva *Catal. Sci. Technol.*, **2022**, 12, 259–27
